# The natural killer cell response to West Nile virus in young and old individuals with or without a prior history of infection

**DOI:** 10.1371/journal.pone.0172625

**Published:** 2017-02-24

**Authors:** Yi Yao, Dara M. Strauss-Albee, Julian Q. Zhou, Anna Malawista, Melissa N. Garcia, Kristy O. Murray, Catherine A. Blish, Ruth R. Montgomery

**Affiliations:** 1 Department of Internal Medicine, Yale University School of Medicine, New Haven, Connecticut, United States of America; 2 Stanford Immunology, Stanford University School of Medicine, Stanford, California, United States of America; 3 Department of Medicine, Stanford University School of Medicine, Stanford, California, United States of America; 4 Department of Pediatrics, Baylor College of Medicine and Texas Children’s Hospital, Houston, Texas, United States of America; 5 Program on Human Translational Immunology, Yale University School of Medicine, New Haven, Connecticut, United States of America; University of Texas Medical Branch at Galveston, UNITED STATES

## Abstract

West Nile virus (WNV) typically leads to asymptomatic infection but can cause severe neuroinvasive disease or death, particularly in the elderly. Innate NK cells play a critical role in antiviral defenses, yet their role in human WNV infection is poorly defined. Here we demonstrate that NK cells mount a robust, polyfunctional response to WNV characterized by cytolytic activity, cytokine and chemokine secretion. This is associated with downregulation of activating NK cell receptors and upregulation of NK cell activating ligands for NKG2D. The NK cell response did not differ between young and old WNV-naïve subjects, but a history of symptomatic infection is associated with more IFN-γ producing NK cell subsets and a significant decline in a specific NK cell subset. This NK repertoire skewing could either contribute to or follow heightened immune pathogenesis from WNV infection, and suggests that NK cells could play an important role in WNV infection in humans.

## Introduction

West Nile virus (WNV) is a mosquito-borne enveloped positive-strand RNA virus belonging to the family Flaviviridae, which includes yellow fever, dengue, and Zika viruses [1, 2]. Since its emergence into the United States in 1999, WNV has spread across North America, South America, and the Caribbean, leading to >41,000 cases in the USA, including 1,753 fatalities [3]. The cumulative incidence of WNV infection may reach 3 million people [1] and no vaccine or targeted antiviral treatment against WNV is available. While the majority of WNV infections are asymptomatic (~80%), some infected patients develop mild symptoms of West Nile fever (~20%), and a small subset (<1%) develop severe neuroinvasive disease, which may include meningitis, encephalitis, acute flaccid paralysis, and death. The most well-defined risk factor for symptomatic WNV infection is age: elderly individuals are more susceptible to severe infection with neurological involvement [2]. Age-related changes in immune responses lead to increased susceptibility to infection and reduced responses to vaccination [4, 5] and dysregulation of responses to WNV [6–10]. In a systems immunology profile of WNV patients, we identified a predictive signature of susceptibility including decreased IL-1β induction following infection with WNV and a significant role for the chemokine CXCL10 [11].

Although the responses of innate immune cells, including macrophages, neutrophils, and dendritic cells, play a critical role in the response to WNV infection in humans [6, 7, 12], the role of innate natural killer (NK) cells in the response to human WNV infection is less clear. NK cells are large granular lymphocytes that specialize in early defense against viral infections and tumor cells mediated by cytokines, chemokines, and direct cytotoxicity. NK cells are triggered by cytokines and a repertoire of invariant activating and inhibitory NK receptors [13–17].

NK cell activity is associated with the control of many viral infections, including cytomegalovirus (CMV), HIV-1, influenza virus, hepatitis C virus, and WNV [18–24]. Infection with CMV dramatically skews the NK cell repertoire both *in vivo* and *in vitro*, driving expansion of a NKG2C^+^CD57^+^ NK cell subset [25–27], and this subset expands in CMV-seropositive individuals infected with hepatitis C, HIV, and hantavirus [28–32]. Whether such clonal-like expansions to specific pathogens occur in other viral infections, including WNV, is unclear. During WNV infection, NK cells may be activated by the interaction between NKp44 and the WNV envelope protein [33], but the responding NK cell subsets have not been identified. Recent studies using mass cytometry (CyTOF) have revealed extraordinary phenotypic and functional diversity in NK cell subsets resulting from combinatorial expression of activating and inhibitory receptors [13, 14, 34–36]. We have undertaken the current study to identify a detailed profile of human NK cells relevant to the response to WNV infection.

## Materials and methods

### Human subjects

Blood samples were obtained with written informed consent under guidelines approved for this study by the Human Investigations Committees of Baylor College of Medicine and Yale University School of Medicine. Donors had no acute illness and took no antibiotics or nonsteroidal anti-inflammatory drugs at the time of sampling [11, 37]. Donors with a history of WNV infection were enrolled in Houston, TX and New Haven, CT including asymptomatic subjects (*n* = 10) and symptomatic subjects (*n* = 12) with mild or severe disease following Centers for Disease Control and Prevention criteria as reported previously [6, 11, 37, 38]. WNV serostatus was assessed by a rapid nucleic acid test at the blood bank or by immunoblot as described [6, 38]. Healthy younger (n = 20) and older (n = 14) donors had no history of WNV infection (self-report confirmed by immunoblotting). All blood samples were collected in cell preparation tubes (CPT tubes, BD Biosciences) and centrifuged within 2 h of collection. All assays used fresh cells. CMV serostatus was determined for all subjects by an anti-CMV IgG ELISA kit (Abcam).

### West Nile virus preparation

All WNV experiments were conducted in a Biosafety Level 3 facility licensed by the State of Connecticut and Yale University. WNV virulent strain CT-2741 was passaged in Vero cells (ATCC CCL-81) cultured in DMEM with 10% FBS, purified by centrifugation, and Viral PFU was quantified by plaque assays. A single batch of WNV was produced as stock and used for the entire study [6].

### Cell isolation, stimulation, and infection

Fresh PBMCs were isolated from CPT tubes [11] and used on the day of isolation. PBMCs were plated at 2 × 10^6^ cells/well in a 96 well plate in medium alone (RPMI 1640), or infected with WNV at a multiplicity of infection (MOI) of 1 for 1 h followed by incubation with RPMI 1640 medium/10% FBS for up to 48 h. Anti-CD107a antibody (conjugated with PE-CF594 or allophycocyanin (APC), S1 Table) was added for the final 4 h of incubation; EDTA (2 mM, American Bioanalytical) was added for the final 10 min.

### Antibodies, flow cytometry, and mass cytometry

For fluorescence flow cytometry, samples were incubated with Live/Dead dye (LIVE/DEAD Fixable Dead Cell Stain Kit, Invitrogen) at room temperature (RT) for 30 min, washed 2x in Ca^2+^- and Mg^2+^-free- DPBS (Gibco^®^ Thermo Fisher). Cells were labeled with surface markers (CD3-BB515, CD14-FITC, CD16-APC H7, CD19-BB515, CD56-PE Cy7, CD57-Pacific Blue) followed by fixation (BD FACS Lyse) and permeabilization (BD FACS Perm II) for 10 min each at RT. Cells were then labeled with antibodies for intracellular markers (IFN-γ-APC, and MIP-1β-PE; S1 Table) for 30 min on ice in the dark and analyzed using a LSRII Flow Cytometer (BD Biosciences). Gating was performed in Flowjo V10 (TreeStar). T cells, B cells and monocytes were excluded by tightly gating on CD3^–^CD19^–^CD14^–^ population [39, 40]. A rare population of CD56^–^CD16^+^ cells, which may include non-classical myeloid cells, was also excluded from the gating of NK cell subsets [41, 42].

For mass cytometry, a single batch of metal-conjugated antibodies were purchased (Fluidigm) or conjugated in house using MaxPar X8 labeling kits according to manufacturer’s instructions (Fluidigm), (S1 Table). PBMCs were labeled exactly as described [24, 35, 43]. Groups of samples (8-12/day) were assessed by the CyTOF2 (Fluidigm) in 10–14 independent experiments using a flow rate of 0.045 ml/min in the presence of EQ Calibration beads (Fluidigm) for normalization. An average of 117,000 ± 42,000 cells (mean ± s.d.) from each sample were acquired and analyzed by CyTOF. Gating was performed in Flowjo V10 and on the Cytobank platform. In addition to excluding T cells (CD3^+^) and B cells (CD19^+^), monocytes were also excluded by gating on CD33^–^CD14^–^ population as well as further eliminating the CD56^–^HLA-DR^+^ population [24].

### RNA isolation and qPCR

Total RNA was extracted using the RNeasy Mini Kit (QIAGEN) and cDNA was synthesized using the iScript cDNA Synthesis Kit (Bio-Rad). Primers for quantitative PCR (qPCR) assays for WNV were used to examine infection efficacy as described [11] and for NK ligands were custom prepared by Yale Oligo Synthesis Resource (S2 Table). Amplification was performed in duplicate runs in a CFX96 Touch^™^ Real-Time PCR Detection System (Bio-Rad) for 50 cycles with annealing temperature at 55°C.

### Data processing and clustering and statistical analysis

All FCS files generated by CyTOF were normalized using Normalizer v0.1 MCR. Clustering was performed at least 3 times using Citrus Version 0.08 (https://github.com/nolanlab/citrus) [44]. Eighteen NK markers were used for clustering including 2B4, CD4, CD7, CD8, CD16, CD56, CD57, CD94, killer-cell immunoglobulin-like receptors (KIRs) KIR2DL1, KIR2DL3, KIR3DL1, LILRB1, NKG2A, NKG2C, NKG2D, NKp30, NKp46, and programmed cell death protein 1 (PD-1). Ten thousand cells were clustered from each sample using the SAM model with 1% minimum cluster size. Significance was inferred for false discovery rate < 1% (q < 0.01).

Statistical analyses were performed with Prism 6 (GraphPad) and the open-source statistical package R (http://www.r-project.org). Fisher exact test or Mann-Whitney U-test was used to compare categorical or continuous variables. Multiple t tests were used for pairwise comparisons of multiple group means using the Holm-Sidak method with alpha = 0.05. Wilcoxon signed-rank test was used for paired data between mock and treatments.

Complete datasets from this study are available through the NIAID ImmPort data repository (SDY58).

## Results

### Human NK cells mount a polyfunctional, time-dependent response to WNV infection *in vitro*

**Fig 1:**
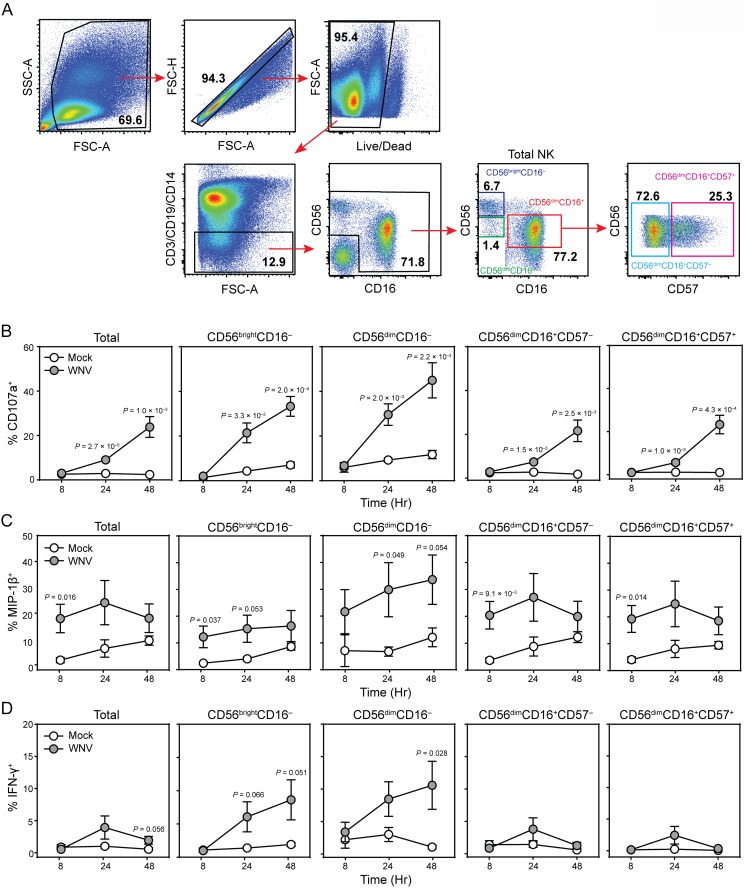
Human NK cell subsets respond to infection with WNV in a time-dependent manner. PBMCs from healthy young subjects (n = 6) were incubated with medium alone (mock) or infected with WNV (MOI = 1) for 8, 24, and 48 h. The samples were labeled with fluorescence-conjugated antibodies against CD3, CD19, CD14, CD56, CD16 and CD57 and analyzed by flow cytometry. (A) Manual gating strategy to define NK cell subsets. Total NK cells, CD56^bright^CD16^-^, CD56^dim^CD16^-^, CD56^dim^CD16^+^CD57^-^, and CD56^dim^CD16^+^CD57^+^ were assessed for surface expression of CD107a (B) and intracellular production of MIP-1β (C) and IFN-γ (D). Means ± s.e.m, multiple t tests.

Recent studies have demonstrated that monoyctes, macrophages and dentritic cells are the major targets of WNV [6, 7, 45]. To assess the NK cell response to WNV infection, we infected PBMCs to best mimic NK cell activation *in vivo*, where multiple cell types can influence the NK cell response. PBMCs from WNV-naive healthy controls were incubated ± WNV; a high viability (>93%) and a physiologic frequency (~6%) of NK cells was maintained in both mock- and WNV-infected cultures (Fig 1A, S1A and S1B Fig). WNV infection resulted in dose-dependent NK cell responses, with a multiplicitiy of infection (MOI) of 0.3 sufficient to induce a potent NK cell response (S2 Fig). In response to WNV infection, the frequency of NK cells expressing the chemokine MIP-1β is significantly increased at 8 hours, CD107a (a marker of degranulation as a surrogate for cytolytic activity) is significantly increased at 24 h and 48 h, and IFN-γ is significantly increased at 48 hours post-infection (Fig 1B–1D).

To identify the subsets responding to WNV infection, we considered the maturation state of the NK cells based on expression of CD56, CD16, and CD57 (Fig 1A). WNV infection is associated with a significantly increased frequency of CD56^bright^CD16^-^ NK cells, possibly reflecting CD16 cleavage or intermediate differentiation [46], but other subsets did not significantly differ in frequency (S1C Fig). While all NK cell subsets demonstrate significant degranulation at 24 and 48 hours post-infection, the highest proportion of responding cells is found within the CD56^bright^CD16^-^ and CD56^dim^CD16^-^ NK cells (Fig 1). In these subsets, MIP-1β-production occurs earlier, and is significantly increased above mock-infected samples at 8 hours and is sustained at 24 and 48 hours. CD56^bright^CD16^-^ and CD56^dim^CD16^-^ subsets are also significantly enriched in IFN-γ producing cells at 24 and 48 hours post-infection. Thus, the most pronounced degranulation, chemokine, and cytokine responses are observed in CD56^bright^CD16^-^ and CD56^dim^CD16^-^ NK cell subsets.

### Phenotypic signature of NK cell receptor and ligands in response to WNV

**Fig 2:**
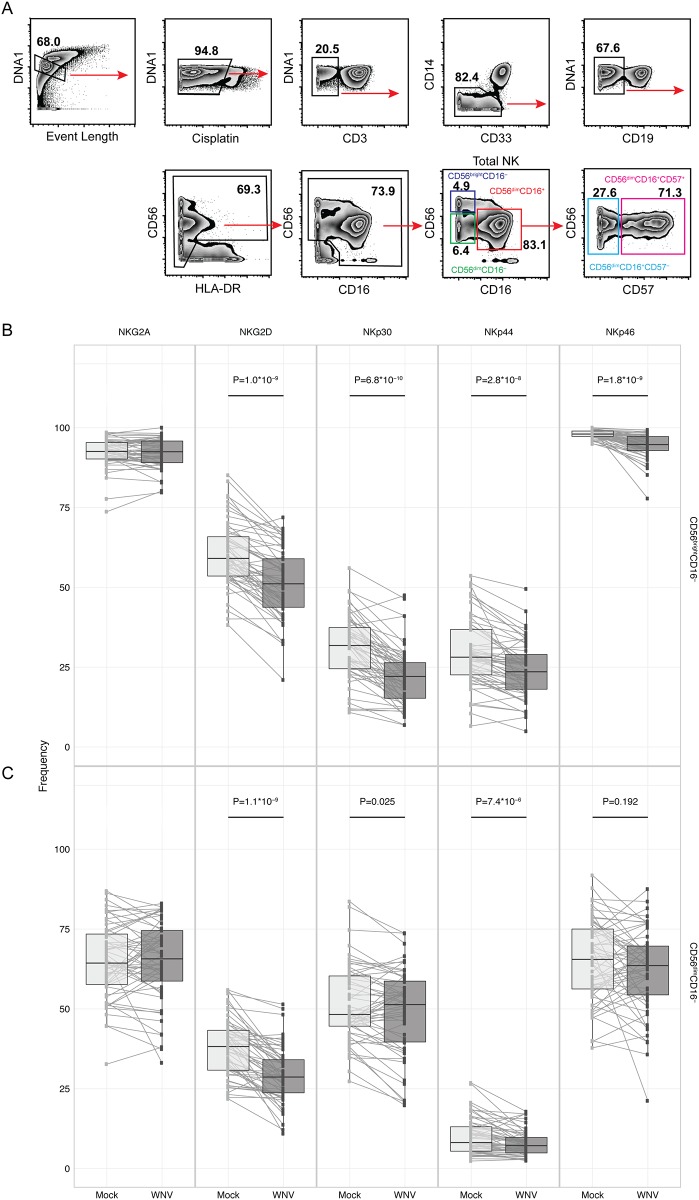
Downregulation of activating receptors in NK cells in response to WNV infection. PBMCs were incubated with medium alone (mock) or infected with WNV (MOI = 1) for 24 h. The samples were labeled with metal-conjugated antibodies and analyzed by mass cytometry. (A) Manual gating strategy to define NK cell subsets. CD56^bright^CD16^-^ (B) and CD56^dim^CD16^-^ (C) NK cell subsets were compared between mock and WNV-infected groups for expression of NK cell inhibitory receptor NKG2A and activating receptors NKG2D, NKp30, NKp44, and NKp46 (n = 56). Paired Wilcoxon tests.

We next used mass cytometry to provide a more precise identification of the phenotypic and functional subsets of NK cells responding to WNV infection (Fig 2A, [35, 36]). We evaluated the WNV responses in PBMCs from 56 individuals including healthy younger (mean age 25) and older (mean age 72) individuals without a history of WNV infection, and healthy individuals with a history of either asymptomatic or symptomatic WNV infection (Table 1). Unlike the dramatic expansion of NKG2C^+^CD57^+^ NK cells observed during the response to CMV infection *in vitro* [26, 27], we did not observe an expansion of a detectable ‘clonal-like’ population of NK cells in response to WNV infection. However, in response to WNV infection, the highly responsive CD56^bright^CD16^-^ and CD56^dim^CD16^-^ NK cell subsets significantly down-regulate expression of the activating receptors NKG2D, NKp30, and NKp44, though other receptors, including NKG2A, do not significantly change in expression level (Fig 2B and 2C). NKp46 is also significantly decreased in the CD56^bright^CD16^-^ population (Fig 2B), and the magnitude of receptor downregulation varies between cohort groups, with the WNV-experienced asymptomatic and symptomatic individuals showing the most dramatic NKG2D and NKp30 down-regulation (S3 Fig). WNV-induced downregulation of these activating receptors could reflect activation-induced internalization.

**Table 1:** Cohort demographics.

Parameter	Young WNV naïve (n = 20)	Old WNV naïve (n = 14)	*P*-value (Young v. old)*	WNV-infected Asymptomatic (n = 10)	WNV-infected Symptomatic (n = 12)	*P* value (asymp vs. symp)
Mean age (yr) (SD), range	25.0 (2.0), 22–30	71.7 (5.7), 66–85	<0.0001	44.8 (22.5), 20–89	51.7 (12.5), 36–71	0.38
No. (%) of females	16 (80.0)	4 (28.6)	0.0046	5 (50.0)	2 (16.7)	0.17
No. (%) of CMV+ subjects	6 (30.0)	6 (42.9)	0.49	5 (50.0)	4 (33.4)	0.67
No. (%) of patients of race						
White	12 (60.0)	13 (92.9)	0.0504	9 (90.0)	11 (91.7)	0.36
Black	0 (0)	1 (7.1)		0 (0)	1 (8.3)	
Asian	7 (35.0)	0 (0)		1 (10.0)	0 (0)	
Other	1 (5.0)	0 (0)				
Hispanic	3 (15.0)	1 (7.1)	0.63	0 (0.0)	1 (8.3)	1.0
Time since infection** yr (SD), range				5.4 (3.2), 0.9–8.8	5.0 (5.0), 0.5–12.0	0.86

*P values were calculated based on a t test for continuous variables and Fisher exact tests for categorical variables.

** For asymptomatic subjects, surrogate onset date is defined as January 1 of the enrollment year

**Fig 3:**
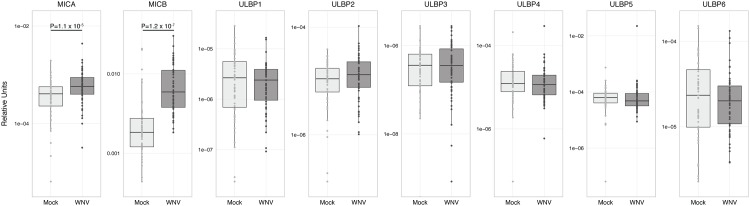
Expression levels of NKG2D ligands in PBMCs with infection of WNV *in vitro*. PBMCs were incubated with medium alone (mock) or infected with WNV (MOI = 1) for 24 h. Samples from all subjects (n = 56) were compared by qPCR between mock and WNV-infected groups for expression of MICA, MICB, ULBP1, ULBP2, ULBP3, ULBP4, ULBP5, and ULBP6, paired Wilcoxon tests.

To investigate this mechanism, we quantified NK receptor ligand expression levels by qPCR, focusing on NKG2D ligands since most of the ligands for the natural cytotoxicity receptors remain unknown [47]. Consistent with the idea that NKG2D could play a role in recognition of WNV-infected cells, the NKG2D ligands MHC class I related chain A (MICA) and MICB are significantly upregulated by WNV infection (Fig 3). However, none of the UL16 binding proteins are significantly changed by WNV infection (Fig 3).

### NK cell responses to WNV infection in young and old individuals

**Fig 4:**
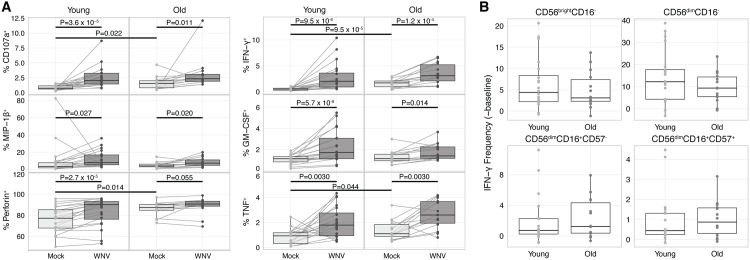
Robust functional response to WNV by NK cells from younger and older healthy subjects. PBMCs from young (n = 20) and old (n = 14) healthy subjects were incubated with medium alone (mock) or infected with WNV (MOI = 1) for 24 h. (A) Total NK cells from mock and WNV-infected groups of each WNV subject were compared by mass cytometry for surface expression of CD107a and production of production of MIP-1β, perforin, IFN-γ, GM-CSF, and TNF. Mock-WNV comparisons, paired Wilcoxon tests. Mock-mock and WNV-WNV comparisons, Mann-Whitney U tests. (B) Four NK cell subsets from young and old healthy subjects were compared for induction level of IFN-γ by subtracting baseline of the mock group from the WNV-infected group. Mann-Whitney U tests.

As aging is strongly associated with severity of WNV infection [2], we compared NK cell responses between young and old WNV naïve individuals (Table 1). NK cells from both age groups mount a significant response to WNV-infection, characterized by degranulation, perforin induction, and cytokine/chemokine production (Fig 4A). While WNV E protein expression levels are higher in younger donors following WNV infection (S4 Fig), there are no significant differences between younger and older donors in frequency of NK cells responsive to WNV infection, despite elevated levels of baseline expression of CD107a, perforin, IFN-γ and TNF in mock-infected samples from older donors (Fig 4A). Even in the highest responding CD56^bright^CD16^-^ and CD56^dim^CD16^-^ NK cells, there are no age-related differences in WNV-induced production of IFN-γ (Fig 4B). As CMV infection can dramatically alter the NK cell repertoire and responsiveness, we also considered potential effects of CMV infection. CMV seropositivity was equally distributed between the cohorts (Table 1), and CMV status did not significantly alter WNV responses (S5 Fig).

As aging is associated with phenotypic skewing of the NK cell repertoire, independent of the effects of CMV infection [21, 48, 49], we used Citrus [44] to identify phenotypic clusters of NK cells that differ in frequency between young and old subjects (S6 Fig). Older individuals have expansions in mature CD56^dim^CD16^+^CD57^+^ subsets with variable expression of NKG2A, PD-1, KIR3DL1, CD94, NKG2D, and other NK cell markers (S6 Fig). However, this phenotypic skewing is not associated with altered functional responses to WNV (Fig 4).

### Subjects with a history of symptomatic WNV infection have an elevated frequency of IFN-γ producing CD56^bright^CD16^-^ and CD56^dim^CD16^-^ NK cells

**Fig 5:**
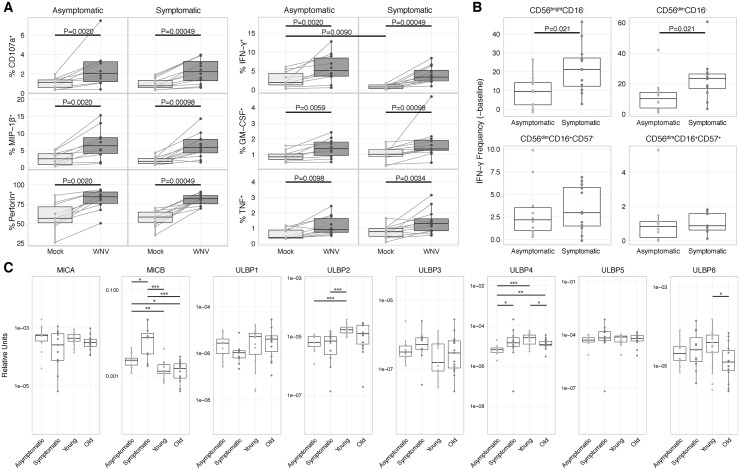
Robust functional response to WNV by NK cells from asymptomatic and symptomatic WNV subjects. PBMCs from asymptomatic (n = 10) and symptomatic (n = 12) WNV subjects were incubated with medium alone (mock) or infected with WNV (MOI = 1) for 24 h. (A) Total NK cells from mock and WNV-infected samples of each WNV subject were compared by mass cytometry for surface expression of CD107a and production of MIP-1β, perforin, IFN-γ, TNF, and GM-CSF. Mock-WNV comparisons, paired Wilcoxon tests. Mock-mock and WNV-WNV comparisons, Mann-Whitney U tests. (B) Four NK cell subsets from asymptomatic and symptomatic WNV subjects were compared for induction of IFN-γ by subtracting baseline of the mock group from the WNV-infected group. Mann-Whitney U tests. (C) Samples from all four groups of subjects (n = 56) were compared by qPCR for baseline expression of MICA, MICB, ULBP1, ULBP2, ULBP3, ULBP4, ULBP5, and ULBP6. Mann-Whitney U tests. P < 0.05 = *; P < 0.01 = **; P < 0.001 = ***.

We examined how a prior history of symptomatic or asymptomatic WNV infection alters the response to *in vitro* WNV infection. There were no significant differences in age, sex, race/ethnicity between the asymptomatic and symptomatic subjects. To minimize the effects of the *in vivo* WNV loads on the NK cell responses in the *in vitro* infection, the subjects with a history of WNV infection were recruited at least 6 months following infection when WNV has been shown to be undetectable [50]. For symptomatic subjects, the interval since infection was 5.0 years (range 0.5–12) and was equivalent to the surrogate infection date of 5.4 years (range 0.9–8.8) (January 1 of the year of enrollment) necessary as the timing of asymptomatic infections could not be determined [11, 37]. As observed with responses from WNV-naive subjects, subjects from both asymptomatic and symptomatic groups demonstrate a robust functional response to WNV infection *in vitro* with significantly increased expression of CD107a, MIP-1β, perforin, IFN-γ, GM-CSF, and TNF (Fig 5A). While symptomatic subjects have 2.5-fold lower levels of baseline (unstimulated) IFN-γ production (p = 0.0090, Fig 5A), WNV-induced responses are not significantly different between the asymptomatic and symptomatic subjects when examining the total NK cell populations. However, IFN-γ production is more than 2-fold enhanced among CD56^bright^CD16^-^ and CD56^dim^CD16^-^ NK cells in symptomatic compared to asymptomatic subjects (p = 0.021 for both cell subsets, Fig 5B). This differential responsiveness is not due to differences in infection levels, as WNV E gene expression levels following infection do not significantly differ between asymptomatic and symptomatic subjects (S4 Fig). Thus, a history of symptomatic WNV infection is associated with significantly elevated IFN-γ response to WNV infection that is not seen with aging alone.

When we compared the baseline expression of NK cell ligands in PBMCs, we identified 3-6-fold upregulation of MICB in symptomatic subjects compared to all other groups (Fig 5C). Symptomatic subjects also have 2.2-fold higher levels of ULBP4 than asymptomatic subjects (p = 0.024, Fig 5C). Expression levels of the remaining NKG2D ligands did not follow a consistent pattern. Thus, symptomatic subjects have significantly higher expression of ligands for NK cell activating receptors that could contribute to enhanced NK cell inflammatory responses.

### Influence of symptomatic WNV infection on the phenotypic NK cell repertoire

**Fig 6:**
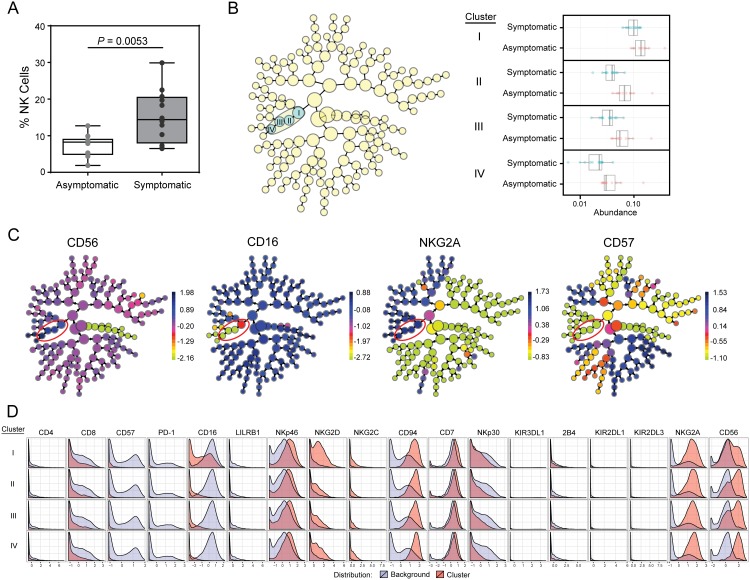
Decreased frequency of immature NK cells in subjects with a history of symptomatic WNV infection. (A) Frequency of total NK cells in asymptomatic (n = 10) and symptomatic (n = 12) WNV subjects. (B-D) The NK dataset from asymptomatic and symptomatic subjects was analyzed by automated hierarchical clustering. Distinguishing clusters between the two groups were identified using SAM model (q < 0.01). (B) Stratifying clusters (yellow circles) including distinguishing clusters between the two groups (blue circles) and abundance of cells within the identified distinguishing clusters. (C) Expression of CD56, CD16, NKG2A and CD57 of stratifying clusters. (D) Histograms to characterize cluster features, with red outline indicating the expression pattern in the cluster that differed between groups and the blue outline indicating the level of staining in the population as a whole.

Symptomatic WNV infection is associated with a significant increase in the frequency of NK cells ((8.2 vs. 14.4%, p = 0.0053); Fig 6A), leading us to examine how symptomatic infection influences the NK cell repertoire. Using Citrus to evaluate NK cell subsets based on expression of 18 surface cell markers, we identified four related phenotypic clusters of NK cells that are significantly higher in frequency in asymptomatic subjects compared to symptomatic subjects with a false discovery rate of 1% (Fig 6B). Three of these clusters are characterized by marker expression patterns indicative of immaturity (CD56^bright^NKG2A^+^CD16^-^CD57^-^), and are also characterized by low expression of CD8, NKp30, LILRB1, and 2B4, and high expression of NKp46, NKG2D and CD94 (Fig 6C and 6D). A fourth cluster has intermediate levels of CD16 but shares the other phenotypic characteristics with the other three (Fig 6C and 6D). A history of symptomatic WNV infection is therefore associated with a higher overall frequency of NK cells, yet a significantly decreased proportion of immature NK cells with a complex pattern of receptor expression, including high expression of CD56, NKG2A, NKp46 and NKG2D but low expression of NKp30, 2B4, CD8, LILRB1, CD16, and CD57.

## Discussion

We used mass cytometry to investigate the phenotype and function of NK cells responding to WNV infection in the setting of aging and prior exposure to WNV infection. This study builds on recent work highlighting the role of diversity in the NK cell repertoire in responding to viruses [24–26, 28–32, 35, 36]. Our data demonstrate that human NK cells mount a robust response to WNV infection *in vitro*, and this response is most prominent among CD56^bright^CD16^-^ and CD56^dim^CD16^-^ NK cells. Further, responding cells downregulate several activating receptors, including NKp30, NKp44, NKp46, and NKG2D, coincident with upregulation of NKG2D ligands in infected cultures, suggesting that these receptors could play a role in recognition of infected cells. Though aging is the most prominent risk factor for symptomatic WNV infection, NK cells from young and old WNV-naïve individuals do not significantly differ in their responses to WNV infection. However, individuals with a history of symptomatic infection display enhanced IFN- γ responses to WNV infection among the highly responsive CD56^bright^CD16^-^ and CD56^dim^CD16^-^ NK cells. Further, individuals with a history of symptomatic infection display a phenotypic skewing of the NK cell repertoire, characterized by a decreased proportion of CD56^bright^CD16^+^NKG2A^+^CD94^+^NKp46^+^NKG2D^+^CD8^-^NKp30^-^LILRB1^-^2B4^-^ NK cells, reflecting WNV-associated shaping of the NK cell repertoire, and suggesting that NK cells could play an important role in WNV pathogenesis in humans.

In the murine model, though NK cells may contribute to the WNV response, they are not necessary, as NK cell depletion does not alter mortality during murine WNV infection [51, 52]. However, there are significant differences in viral evasion mechanisms and immune regulatory pathways between human and mouse [53], so the possibility remains that NK cells may play a role in human infection with WNV. Notably, mice are not within the natural host range of WNV, and murine NK cells may not have evolved to respond to WNV infection. In fact, unlike murine NK cells, human NK cells are activated following interaction of the NKp44 receptor with WNV envelope protein [23, 33]. While we cannot prove causation in this human study, our data suggest that specific subsets of human NK cells can mount a robust response to WNV infection, resulting in cytolytic activity, cytokine secretion, and chemokine secretion that is capable of influencing the quality and the quantity of the immune response to WNV infection in humans.

Interestingly, although aging is the best characterized risk-factor for disease severity, the nature of this response is not significantly different between young and old individuals. Consistent with prior studies [49, 54, 55], we observe a dramatic phenotypic skewing in aged individuals, most prominently characterized by an expansion of CD57-expressing NK cells. Furthermore, CMV infection, though it is associated with expansion of NKG2C^+^CD57^+^ NK cells in both young and old individuals [21, 25, 26], is not associated in alterations in the quality and quantity of the NK cell response to WNV in this study. CMV may do little to alter the response to WNV because the WNV responses are most prominent in the CD56^bright^CD16^-^ and CD56^dim^CD16^-^ NK cell subsets that are generally CD57^-^. It should be noted that subjects recruited in this study had relatively low average CMV seropositivity (37.5%) compared to the overall (50.4%) age-adjusted CMV seroprevalence in the USA [56]. Any of the genetic and social-environmental factors associated with CMV seropositivity, such as age, gender, ethnicity, and levels of household income, insurance and education [56], may contribute to this difference due to relatively small sample size in the current study.

In contrast to the relatively unaltered responses with aging, individuals with a history of symptomatic infection have a higher frequency of IFN-γ producing CD56^bright^CD16^-^ and CD56^dim^CD16^-^ NK cells in response to WNV infection. Our observational study cannot distinguish whether this is a consequence of prior symptomatic infection or an intrinsic characteristic in a subset of individuals that contributes to heightened pathogenesis upon primary infection. An elevated cytokine response to WNV infection promotes permeability of the blood brain barrier and viral penetration of the brain in the murine model [57], suggesting that hyperinflammatory responses could be counterproductive *in vivo*. The fact that symptomatic infection is also associated with a significant skewing in the NK cell repertoire suggests that an interaction between NK cells and WNV infection could alter the repertoire during symptomatic infection *in vivo*, consistent with the enhanced diversification associated with immune experience and viral exposure we noted in an earlier study [36].

Limitations of our study include our inability to severity-stratify responses during acute infection as asymptomatic subjects are largely unaware of their infection [38] and the modest subject numbers, which may have prevented us from finding additional signatures associated with WNV infection. In addition, the mechanisms by which NK cells are activated during WNV infection remain to be determined. We evaluated WNV infection in mixed PBMC infection to maximize discovery from the patient samples—which are limited in volume—and to better model the *in vivo* interactions in which NK cell activation can be influenced by multiple cell types. However, this limits our ability to define the mechanisms by which specific NK cell receptor:ligand pairs contribute to the WNV response. While use of a specific target cell may enhance the infection efficiency since more target cells would be infected, the losses through purification would substantially limit our ability to track sufficient numbers of NK cells. In particular, while our data suggest that natural cytotoxicity receptors and NKG2D could play a role in WNV recognition, NKG2D is also expressed on CD8^+^ T cells, and the upregulation of NKG2D ligands on infected cells may target recognition by more than one immune cell type [51]. In addition to the direct interaction between NK receptors and their ligands, NK cell activation can also be triggered by type I interferons [58–60], which are abundantly produced by WNV infected macrophages and dendritic cells [6, 7, 61]. Moreover, UV-inactivated WNV is sufficient to induce CD107a surface expression by an NKp44 receptor-expressing cell line, suggesting that WNV replication may not be required for NK cell response to WNV [33]. Thus the present studies can not differentiate direct effects of infection from the effects of bystander un-infected cells, which may be significant for the magnitude of the response by activating NK cells.

In conclusion, human NK cells mount a robust, polyfunctional cell response to WNV infection characterized by cytolytic activity, cytokine, and chemokine secretion. The heightened functional response to WNV infection in individuals with a history of symptomatic infection could reflect past exposure, akin to the skewing observed with CMV infection, or play a role in the pathogenesis of symptomatic WNV infection. Combined with recent data indicating that individuals with neuroinvasive WNV infection have expansions of WNV-specific T cells that are skewed towards production of IFN-γ and IL-4 [62], these data suggest that immune-mediated pathogenesis from multiple cell types may contribute to severe outcomes in WNV infection. Effective control of WNV infection may therefore rely on addressing this overly exuberant inflammatory response.

## Supporting information

S1 Fig Cell viability and NK cell frequency following infection with WNV over 48 h time course. PBMCs from healthy young subjects (n = 6) were incubated with medium alone (mock) or infected with WNV (MOI = 1) for 8, 24, and 48 h as in Fig 2. (A) Viability of PBMCs at indicated time points for both mock and WNV-infected groups. (B) Frequency of total NK cells in PBMCs. (C) Frequency of NK cell subsets in total NK cells. Multiple t tests. Error bars indicate means ± s.e.m.
(DOCX)

S2 Fig Human NK cell subsets respond to infection with WNV in a dose-dependent fashion. PBMCs from healthy young subjects (n = 4) were incubated with medium alone (mock) or infected with WNV at MOI as 0.3,1, 3 and 10 for 24 h. The samples were labeled with fluorescence-conjugated antibodies against CD3, CD19, CD14, CD56, CD16, CD57, CD107a, MIP-1β and IFN-γ and analyzed by flow cytometry. Error bars indicate means ± s.e.m. *P < 0.05.
(DOCX)

S3 Fig Downregulation of activating receptors in NK cells in response to WNV infection in subjects with a history of WNV infection. PBMCs were incubated with medium alone (mock, light grey) or infected with WNV (MOI = 1, dark grey) for 24 h. CD56^bright^CD16^-^ and CD56^dim^CD16^-^ NK cell subsets were compared between mock and WNV-infected groups for expression of NK activating receptors NKG2D, NKp30, NKp44, and NKp46 (n = 56). Paired Wilcoxon tests.
(DOCX)

S4 Fig WNV viral load in PBMCs with infection of WNV *in vitro*. PBMCs from asymptomatic (n = 10), symptomatic (n = 11), young (n = 15), and old (n = 14) subjects were incubated with medium alone (mock) or infected with WNV (MOI = 1) for 24 h. Expression of WNV *E*-gene mRNA was quantified by qPCR from samples of all subjects. *P < 0.05.
(DOCX)

S5 Fig Effect of CMV status on NK cell functionality. All subjects (n = 56) recruited in this study were screened for CMV serotypes by ELISA. PBMCs from all subjects were infected with WNV as in Figs 3 and 5. Total NK cells within CMV^+^ or CMV^-^ groups were compared at baseline and following infection with WNV for surface expression of CD107a and production of perforin, IFN-γ, MIP-1β, GM-CSF and TNF by mass cytometry. ***P < 0.001; ****P < 0.0001; N.S. not significant.
(DOCX)

S6 Fig Increased frequency of mature NK cells in healthy older subjects. (A) Frequency of total NK cells in young (n = 20) and old (n = 14) healthy subjects. (B-D) The NK dataset from young (n = 20) and old (n = 14) healthy subjects was analyzed by automated hierarchical clustering. (B) Stratifying clusters (yellow circles) including distinguishing clusters between the two groups (blue circles) and abundance of cells within the identified distinguishing clusters. (C) Expression of CD56, CD16, NKG2A and CD57 of stratifying clusters. (D) The phenotypic plots represent the clusters with different abundance between the younger and older subjects. All the phenotypic plots are representative of at least three independent runs.
(DOCX)

S1 Table Antibodies used for flow cytometry and mass cytometry. (DOCX)

S2 Table Sequences for all the primers used for qPCR. (DOCX)

## Abbreviations

APC: allophycocyanin
CyTOF: mass cytometry
KIR: killer-cell immunoglobulin-like receptor
MIC: Major Histocompatibility Complex Class I Chain-Related Protein
MOI: multiplicity of infection
NCRs: natural cytotoxicity receptors
PD-1: programmed cell death protein 1
qPCR: quantitative PCR
ULBP: UL16 binding protein
WNV: West Nile virus
