# Supplementary figures and images for: The natural killer cell response to West Nile virus in young and old individuals with or without a prior history of infection
Source: PLoS One. 2017 Feb 24;12(2):e0172625. doi: 10.1371/journal.pone.0172625 (PMC5325267; doi:10.1371/journal.pone.0172625)

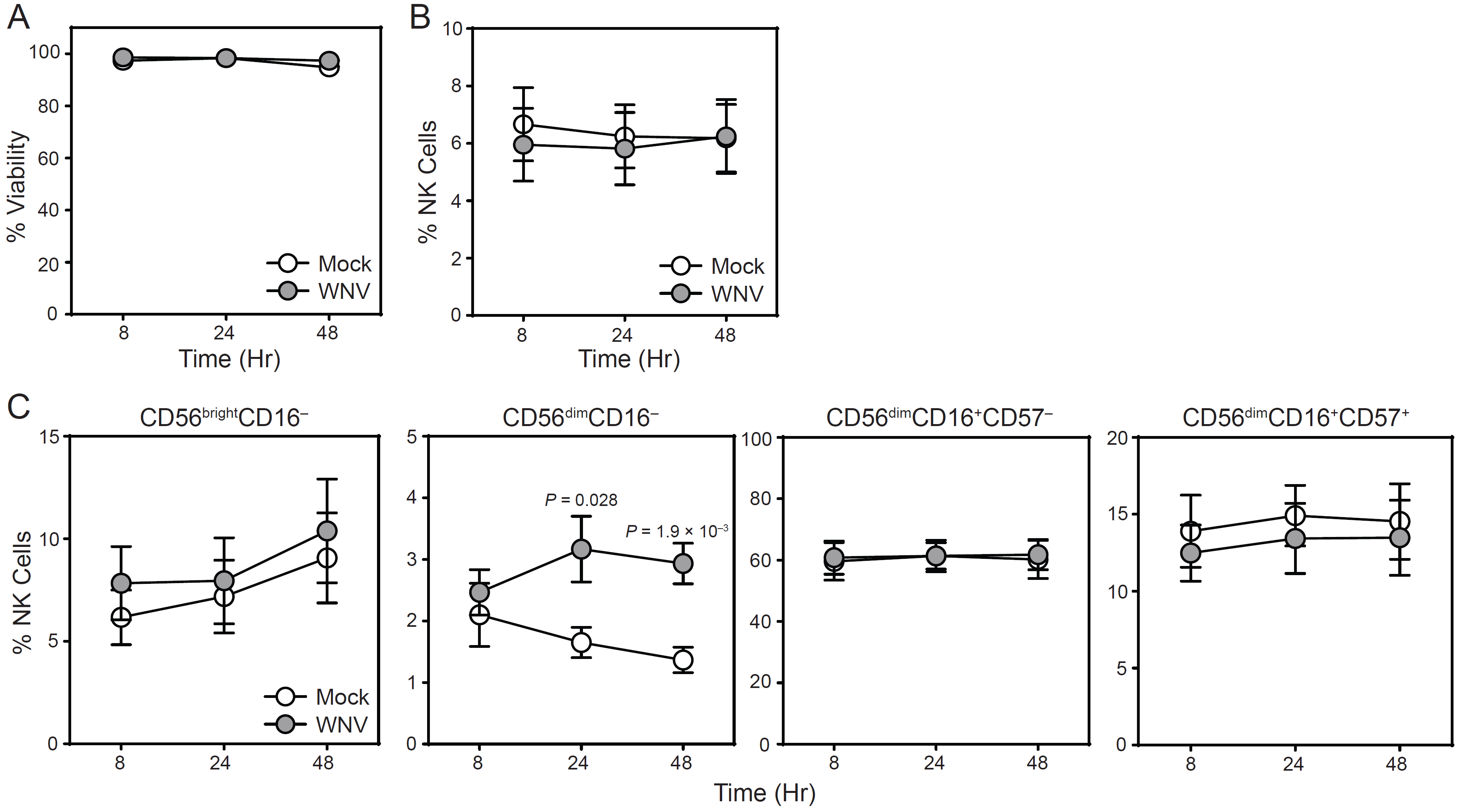


**S1 Fig. Cell viability and NK cell frequency following infection with WNV over 48 h time course.**

Supplement: S1 Fig — PBMCs from healthy young subjects (n = 6) were incubated with medium alone (mock) or infected with WNV (MOI = 1) for 8, 24, and 48 h as in Fig 2. (A) Viability of PBMCs at indicated time points for both mock and WNV-infected groups. (B) Frequency of total NK cells in PBMCs. (C) Frequency of NK cell subsets in total NK cells. Multiple t tests. Error bars indicate means ± s.e.m. (DOCX) [file pone.0172625.s001.docx]

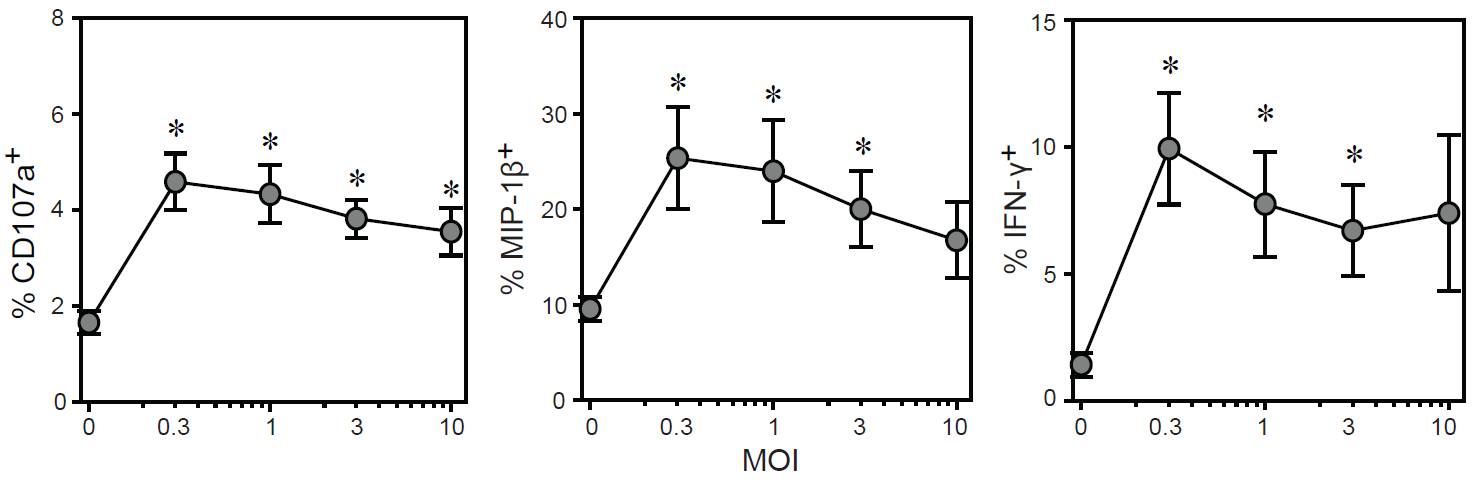


**S2 Fig. Human NK cell subsets respond to infection with WNV in a dose-dependent fashion.**

Supplement: S2 Fig — PBMCs from healthy young subjects (n = 4) were incubated with medium alone (mock) or infected with WNV at MOI as 0.3,1, 3 and 10 for 24 h. The samples were labeled with fluorescence-conjugated antibodies against CD3, CD19, CD14, CD56, CD16, CD57, CD107a, MIP-1β and IFN-γ and analyzed by flow cytometry. Error bars indicate means ± s.e.m. *P < 0.05. (DOCX) [file pone.0172625.s002.docx]

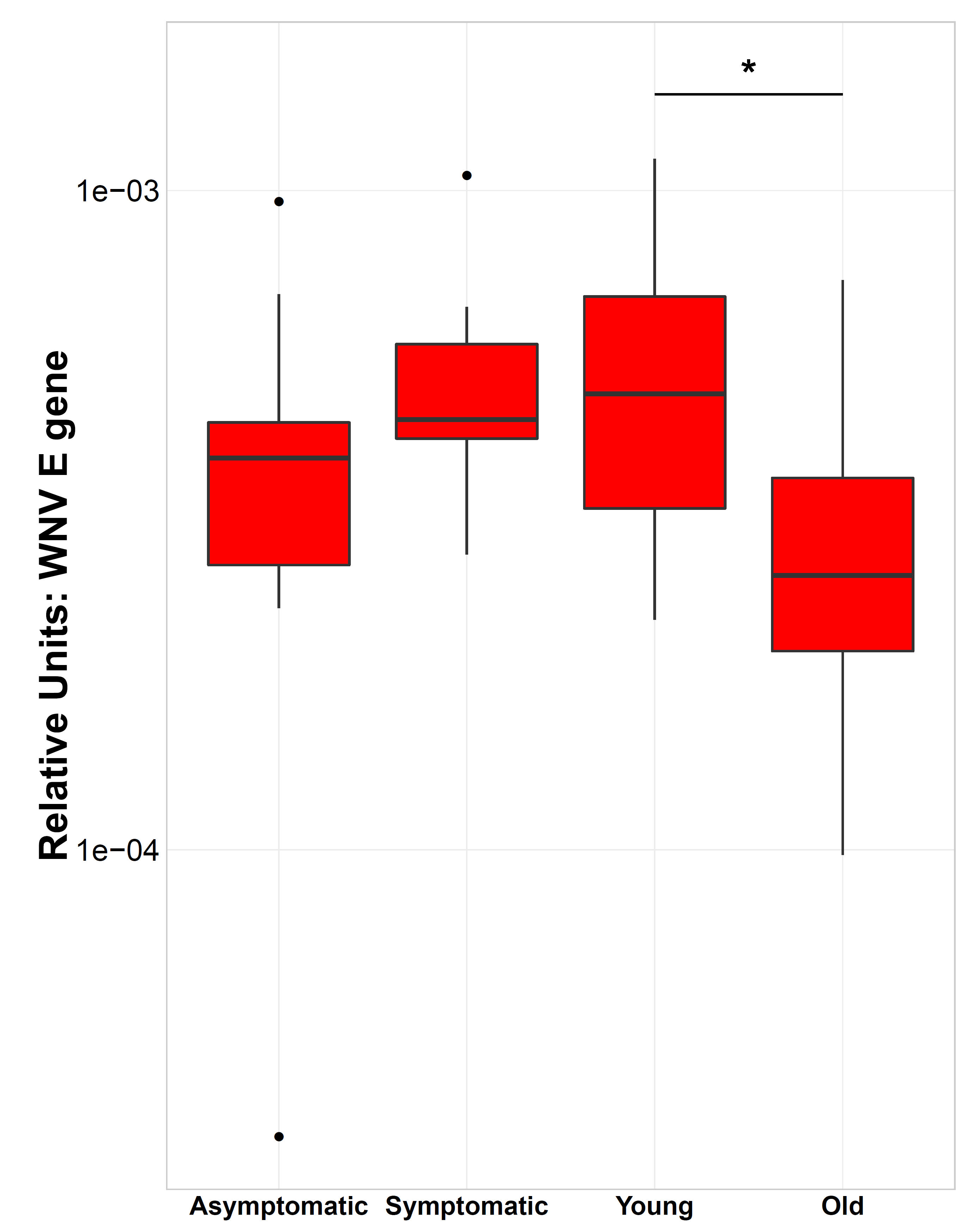


**S4 Fig. WNV viral load in PBMCs with infection of WNV *in vitro*.**

Supplement: S4 Fig — PBMCs from asymptomatic (n = 10), symptomatic (n = 11), young (n = 15), and old (n = 14) subjects were incubated with medium alone (mock) or infected with WNV (MOI = 1) for 24 h. Expression of WNV E-gene mRNA was quantified by qPCR from samples of all subjects. *P < 0.05. (DOCX) [file pone.0172625.s004.docx]

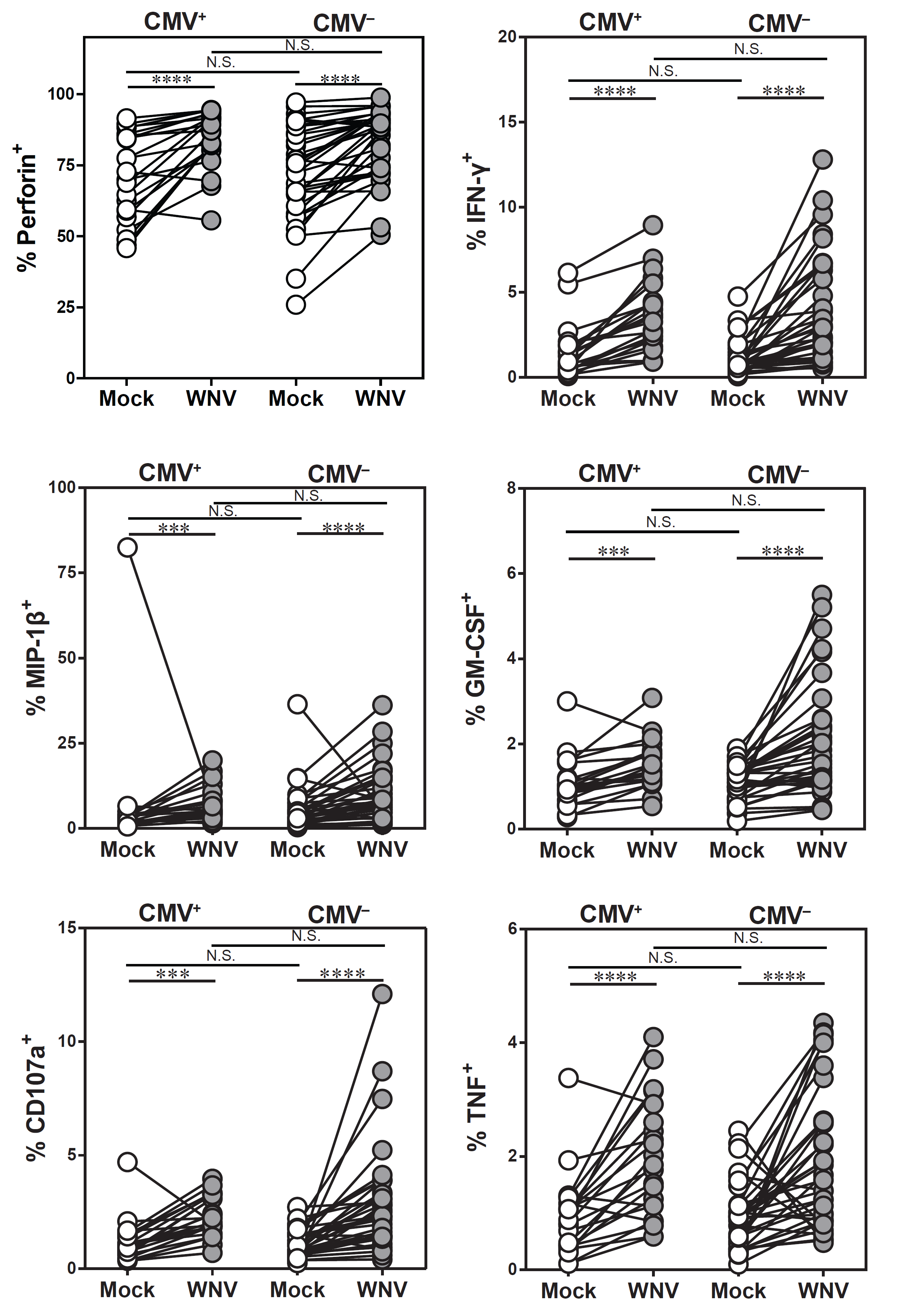


**S5 Fig. Effect of CMV status on NK cell functionality.**

Supplement: S5 Fig — All subjects (n = 56) recruited in this study were screened for CMV serotypes by ELISA. PBMCs from all subjects were infected with WNV as in Figs 3 and 5. Total NK cells within CMV+ or CMV- groups were compared at baseline and following infection with WNV for surface expression of CD107a and production of perforin, IFN-γ, MIP-1β, GM-CSF and TNF by mass cytometry. ***P < 0.001; ****P < 0.0001; N.S. not significant. (DOCX) [file pone.0172625.s005.docx]

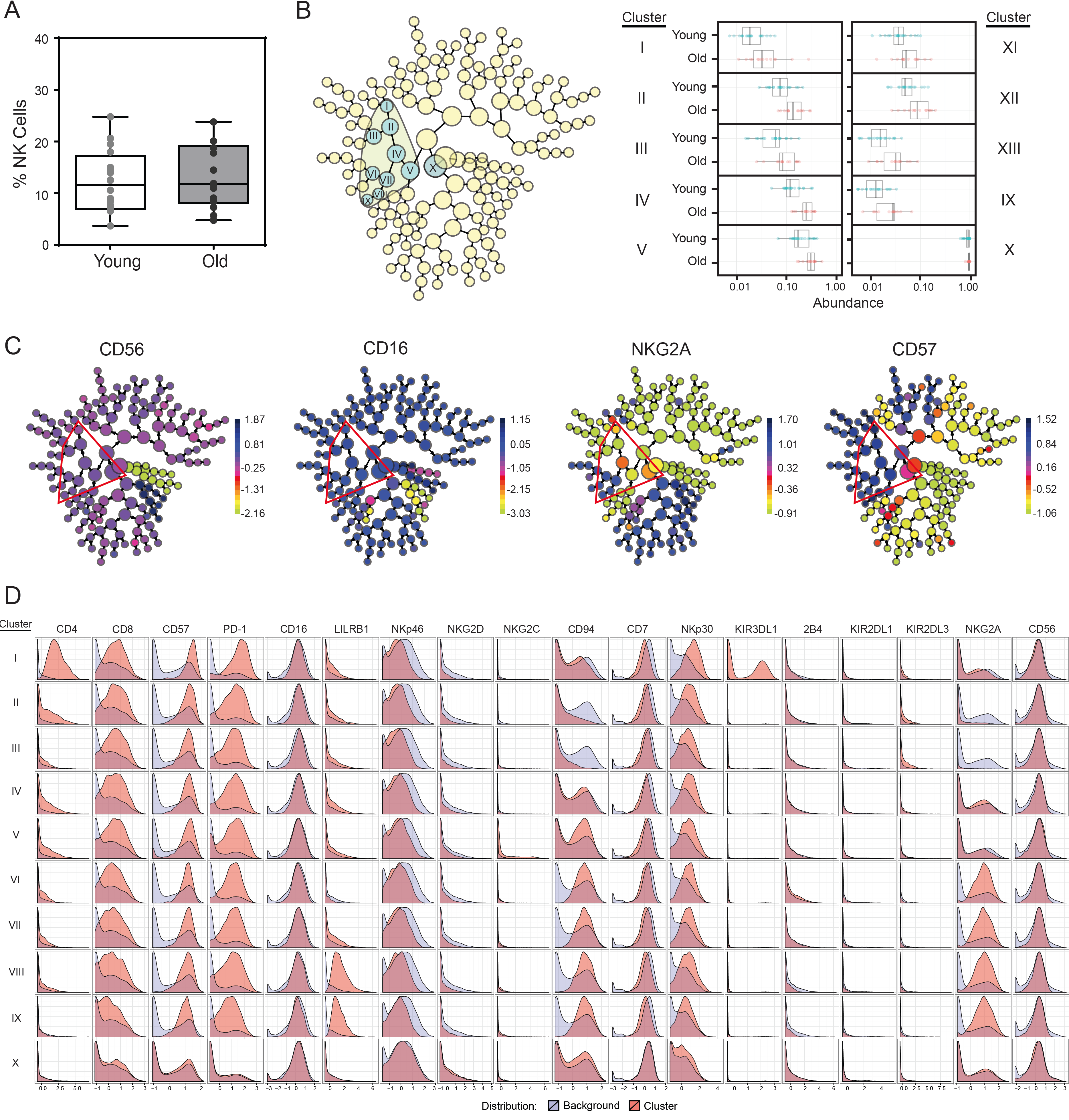


**S6 Fig. Increased frequency of mature NK cells in healthy older subjects.**

Supplement: S6 Fig — (A) Frequency of total NK cells in young (n = 20) and old (n = 14) healthy subjects. (B-D) The NK dataset from young (n = 20) and old (n = 14) healthy subjects was analyzed by automated hierarchical clustering. (B) Stratifying clusters (yellow circles) including distinguishing clusters between the two groups (blue circles) and abundance of cells within the identified distinguishing clusters. (C) Expression of CD56, CD16, NKG2A and CD57 of stratifying clusters. (D) The phenotypic plots represent the clusters with different abundance between the younger and older subjects. All the phenotypic plots are representative of at least three independent runs. (DOCX) [file pone.0172625.s006.docx]
