# Supplementary material for: The natural killer cell response to West Nile virus in young and old individuals with or without a prior history of infection
Source: PLoS One. 2017 Feb 24;12(2):e0172625. doi: 10.1371/journal.pone.0172625 (PMC5325267; doi:10.1371/journal.pone.0172625)
Supplement: S3 Fig — PBMCs were incubated with medium alone (mock, light grey) or infected with WNV (MOI = 1, dark grey) for 24 h. CD56brightCD16- and CD56dimCD16- NK cell subsets were compared between mock and WNV-infected groups for expression of NK activating receptors NKG2D, NKp30, NKp44, and NKp46 (n = 56). Paired Wilcoxon tests. (DOCX) [file pone.0172625.s003.docx]

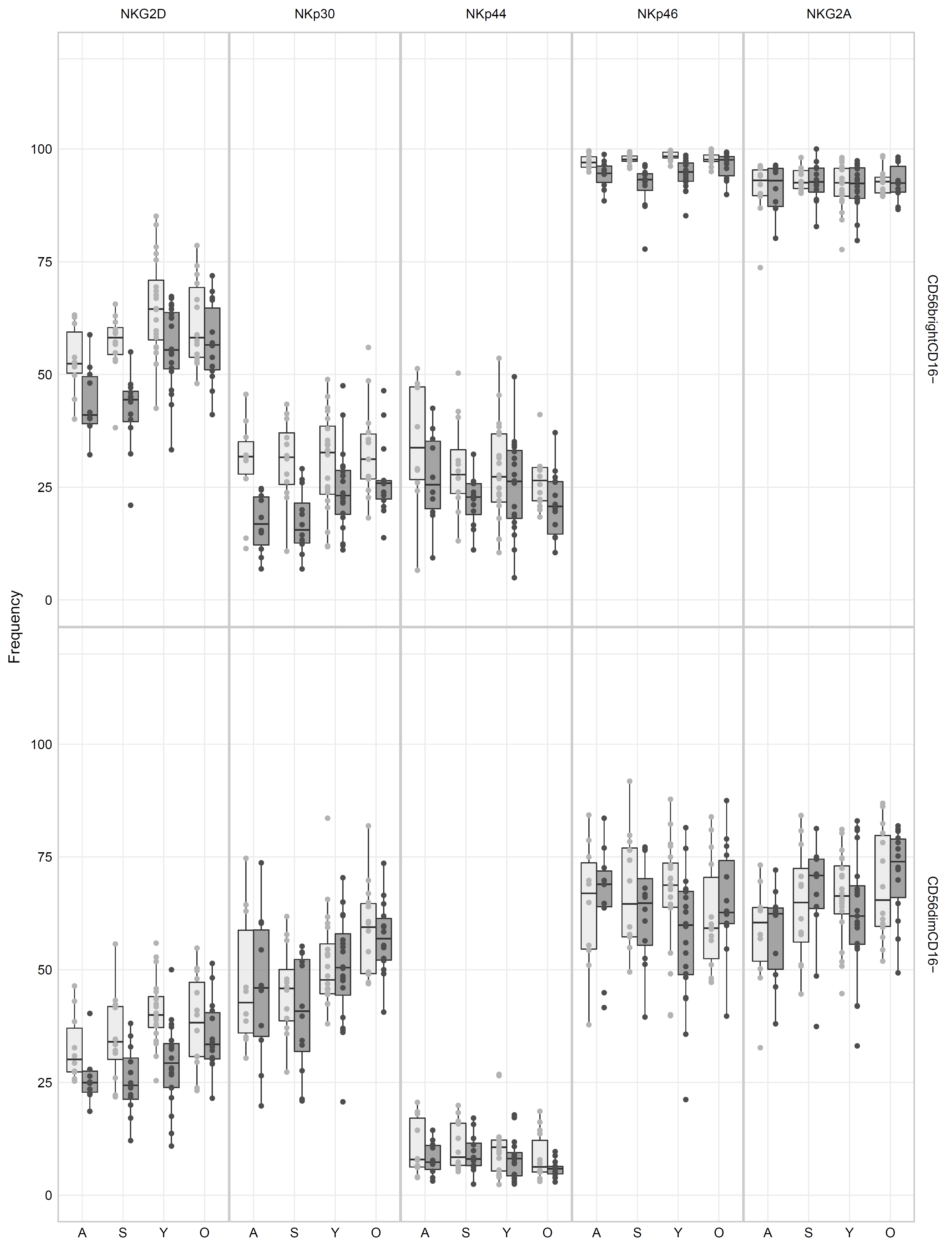


**S3 Fig. Downregulation of activating receptors in NK cells in response to WNV infection in subjects with a history of WNV infection.**
