# Supplementary material for: The natural killer cell response to West Nile virus in young and old individuals with or without a prior history of infection
Source: PLoS One. 2017 Feb 24;12(2):e0172625. doi: 10.1371/journal.pone.0172625 (PMC5325267; doi:10.1371/journal.pone.0172625)
Supplement: S1 Table — (DOCX) [file pone.0172625.s007.docx]

**S1 Table. Antibodies used for flow cytometry and mass cytometry.**

| Fluorophore/Isotope | Ab | Vendor | Catalog # | Clone |
| --- | --- | --- | --- | --- |
| PE-CF594 | CD107a | BD | 562628 | H4A3 |
| APC | CD107a | BioLegend | 328620 | H4A3 |
| BB515 | CD3 | BD | 564465 | UCHT1 |
| FITC | CD14 | eBioscience | 11-0149-42 | 61D3 |
| APC-H7 | CD16 | BD | 557758 | 3G8 |
| BB515 | CD19 | BD | 564456 | H1B19 |
| PE-Cy7 | CD56 | BD | 557747 | B159 |
| Pacific Blue | CD57 | BioLegend | 322316 | HCD57 |
| APC | IFNγ | BD | 554702 | B27 |
| PE | MIP-1β | BD | 550078 | D21-1351 |
| Qdot - Cd | HLA-DR | Life Technologies | Q22158 | TÜ36 |
| 141Pr | Anti-APC (CD107a) | Biolegend | APC003 | 408002 |
| 142Nd | DNAM-1 | BD | 559787 | DX11 |
| 143Nd | CD4 | Biolegend | 344602 | SK3 |
| 144Nd | CD8 | Biolegend | 344702 | SK1 |
| 145Nd | CD57(IgM) | Biolegend | 322302 | HCD57 |
| 146Nd | PD-1 | Biolegend | 329912 | EH12.2H7 |
| 147Sm | CD3 | Biolegend | 300414 | UCHT1 |
| 149Sm | CD16 | BD Pharmingen | 556617 | 3G8 |
| 150Nd | MIP-1β | BD custom | NA | D21-1352 |
| 151Eu | GM-CSF | Biolegend | 502302 | BVD2-21C11 |
| 152Sm | TNF-α | eBioscience | 14-7349-85 | MAb11 |
| 153Eu | KIR2DS4 | Beckman Coulter | IM3337 | FES172 |
| 154Sm | LILRB1 | Beckman Coulter | A07408 | HP-F1 |
| 155Gd | NKp46 | BD Pharmingen | 557911 | 9E2/NKp46 |
| 156Gd | NKG2D | BD Pharmingen | 552866 | 1D11 |
| 157Gd | NKG2C | R&D System | MAB1381 | 134591 |
| 158Gd | CD94 | Biolegend | 305502 | DX22 |
| 159Tb | CD7 | Biolegend | 343102 | CD7-6B7 |
| 160Gd | CD69 | BD Pharmingen | 555529 | FN50 |
| 161Dy | NKp30 | BioLegend | 325202 | P30-15 |
| 162Dy | CD33 | Biolegend | 303402 | WM53 |
| 163Dy | KIR3DL1 | BD Pharmingen | 555964 | DX9 |
| 165Ho | 2B4 | BD Pharmingen | 550814 | 2-69 |
| 166Er | KIR2DL1 | R&D Systems | MAB1844 | 143211 |
| 167Er | Perforin | Abcam | ab47225 | B-D48 |
| 168Er | CD19 | Biolegend | 302214 | HIB19 |
| 169Tm | NKp44 | BioLegend | 325102 | P44-8 |
| 170Er | KIR2DL3 | R&D Systems | MAB2014 | 180701 |
| 171Yb | NKG2A | Beckman Coulter | IM2750 | Z199 |
| 172Yb | IL-10 | BD Pharmingen | 554497 | JES3-9D7 |
| 173Yb | CD14 | Biolegend | 301802 | M5E2 |
| 174Yb | CD56 | BD | 559043 | NCAM16.2 |
| 175Lu | IL-17A | Biolegend | 512302 | BL168 |
| 176Yb | IFN-γ | eBioscience | 14-7319-85 | 4S.B4 |
