# Supplementary material for: The natural killer cell response to West Nile virus in young and old individuals with or without a prior history of infection
Source: PLoS One. 2017 Feb 24;12(2):e0172625. doi: 10.1371/journal.pone.0172625 (PMC5325267; doi:10.1371/journal.pone.0172625)
Supplement: S2 Table — (DOCX) [file pone.0172625.s008.docx]

**S2 Table. Sequences for all the primers used for qPCR**

| **Gene Name** | **Forward** | **Reverse** |
| --- | --- | --- |
| β-actin | ATCCTGGCCTCGCTGTCCAC | GGGCCGGACTCGTCATAC |
| WNV E | TTCTCGAAGGCG ACAGCTG | CCGCCTCCATATTCATCATC |
| MICA | CTGGCTGGCATCTTCCCTTTT | CTCCTGGTGCTGTTGTCTTC |
| MICB | TCTCACCAGCACTTTCCCTCT | TCTTCCACAGCCCTTCGTATTT |
| ULBP1 | GATGGGTCGACACACACTG | AGAGGGTGGTTTTGTTGGA |
| ULBP2 | CCTAGCGCTCTGGGTCC | GTCAAAGAGGAGGAAGAACTGC |
| ULBP3 | GCGATTCTTCCGTACCTGCTA | TGGTGGCTATGGCTTTGGGTT |
| ULBP4 | CCTCAGTTGTTCCAGGGTAAAG | CACATTCACCCTCACTGGTATG |
| ULBP5 | CATCACCGTCATCCCTAAGTTC | CTTGCTGCCACAGTCATAGT |
| ULBP6 | TGGACAGACCTTCCTACTCTT | GGCCACATCCTTGTCATTCT |
